# Optimal assignment methods for ligand-based virtual screening

**DOI:** 10.1186/1758-2946-1-14

**Published:** 2009-08-25

**Authors:** Andreas Jahn, Georg Hinselmann, Nikolas Fechner, Andreas Zell

**Affiliations:** 1 University of Tübingen, Center for Bioinformatics Tübingen (ZBIT), Sand 1, 72076 Tübingen, Germany

## Abstract

**Background:**

Ligand-based virtual screening experiments are an important task in the early drug discovery stage. An ambitious aim in each experiment is to disclose active structures based on new scaffolds. To perform these "scaffold-hoppings" for individual problems and targets, a plethora of different similarity methods based on diverse techniques were published in the last years. The optimal assignment approach on molecular graphs, a successful method in the field of quantitative structure-activity relationships, has not been tested as a ligand-based virtual screening method so far.

**Results:**

We evaluated two already published and two new optimal assignment methods on various data sets. To emphasize the "scaffold-hopping" ability, we used the information of chemotype clustering analyses in our evaluation metrics. Comparisons with literature results show an improved early recognition performance and comparable results over the complete data set. A new method based on two different assignment steps shows an increased "scaffold-hopping" behavior together with a good early recognition performance.

**Conclusion:**

The presented methods show a good combination of chemotype discovery and enrichment of active structures. Additionally, the optimal assignment on molecular graphs has the advantage to investigate and interpret the mappings, allowing precise modifications of internal parameters of the similarity measure for specific targets. All methods have low computation times which make them applicable to screen large data sets.

## Background

Virtual screening (VS) approaches are a fundamental part of the drug discovery pipeline [1,2]. The top-ranked structures of VS experiments are further analysed in biological assays to elucidate their activities. The methods for this purpose can be divided into structure-based and ligand-based VS techniques. Structure-based approaches, like DOCK [3,4] or PLANTS [5], try to dock the ligand into the binding pocket of a target protein, which requires X-ray diffraction or nuclear magnetic resonance spectroscopy experiments to obtain three-dimensional coordinates of the protein structure. Additionally, there are doubts about the ability of docking approaches to predict the affinity or even the rank of the structures [6]. In spite of these negative examples, there exists a remarkable list of successful structure-based VS stories [7].

Ligand-based methods operate only on one or several known active ligands. Therefore, ligand-based approaches are the method of choice if no protein structure is available. The underlying assumption of ligand-based approach is that similar structures have similar biological activity. Maggiora and Johnson [8] introduced the similarity-property principle that implies that the chemical similarity can be related to the biological activity of structures. Although Martin et al. [9] reported that the correlation between structural similarity and biological activity is not so strong, the correlation between activity and similarity is of course the result of the used similarity measure and varies between different methods. Thus, a wide variety of different similarity measures have been proposed in recent years [10,11]. All methods can be divided into different classes concerning the type of information used to calculate the similarity between two structures. A simple and fast approach is to encode the information in fingerprints based on features that are included in the structure. Those topological or structural fingerprints, like the MACCS keys [12] or Daylight fingerprints [13], are often used but lack the ability to perform "scaffold-hoppings" [14]. A more elaborate approach is based on molecular graphs in combination with maximum common substructure or maximum common edge subgraph isomorphism as used by the RASCAL algorithm or different reduced graph approaches [15-17]. Feature Trees are another reduced graph method which uses a reduced tree representation of important molecular fragments like hydrophobic parts or functional groups [18]. The topological based MOLPRINT2D approach uses count vectors of atom types in different layers as molecular atom environment fingerprints and has shown to be useful descriptors for naïve Bayesian classifiers in VS experiments [19].

Similarity measures, based on three-dimensional coordinates, include geometrical information of arbitrary objects defined on structures. The type of an object varies between different methods and can be classified into three types: pharmacophores, molecular shapes or volumes, and molecular (interaction) fields. Pharmacophore based methods generate patterns of distances between predefined molecular properties like aromatic systems or hydrogen bond acceptors/donors [20,21] and calculate a similarity value by a comparison of the corresponding patterns. Molecular shape or volume approaches try to maximize the overlap of shapes or volumes and determine a similarity value based on the overlap. Ballester et al. introduced a non-superposition comparison algorithm for molecular shapes, called Ultrafast Shape Recognition, and applied it in VS experiments [22]. ROCS uses three-dimensional Gaussian functions to describe the volume of query structures and to screen databases in search of structures with similar shapes [23]. Molecular interaction field methods like GRID [24] or CoMFA [25] were originally introduced in the field of quantitative structure-activity relationship modelling (QSAR) but are also suitable for VS experiments [26]. FieldScreen, a recently published method by Cheeseright et al. [27], uses four different types of molecular fields and reduces these fields to the local maximal values. These values, referred to as "field points" [28], serve as the descriptors. This method has shown an improved "scaffold-hopping" behavior on various data sets and improved enrichment rates in comparison to DOCK.

The concept of an optimal assignment of arbitrary objects of molecular graphs was introduced in the field of cheminformatics by Fröhlich et al. [29,30]. Just like CoMFA studies [25,31], the optimal assignment on molecular graphs was used to derive QSAR and quantitative structure-property relationship models. Despite several proofs of the usability of this concept on several data sets and different problems [29,30,32-34], no experiments have been conducted to evaluate this idea in the field of ligand-based VS.

There are two objectives of this study. First, we want to evaluate the existing optimal assignment similarity measure and its flexibility extension on a set of different ligand-based VS experiments. Second, we introduce two new methods based on the optimal assignments. The experiments were designed with emphasis on the evaluation of the "scaffold-hopping" behavior of the methods. This was achieved by using recently suggested VS metrics based on chemotype clustering and extended receiver operating characteristics [35-41]. To benchmark the optimal assignment methods, we compared the results with FieldScreen, the 166 bit MACCS keys and DOCK.

The results show an improved early recognition performance for the optimal assignment methods in comparison to selected literature methods. The performance over the complete data set suffers from late retrievals of dissimilar chemotypes reducing the arithmetic weighted version of the area under the receiver operating characteristic curve values. One of the optimal assignment methods, which is based on two different assignment steps, shows an increased performance on both types of evaluation. The presented methods have low computation times; consequently, they can be applied to screen large databases.

## Methods

In this section we first summarize the optimal assignment problem and give a general definition of the problem. Afterwards, a detailed description of the different methods which are evaluated in the scope of ligand-based VS, follows.

The optimal assignment problem is one of the basic discrete optimization problems. The fundamental task is to find the set of assignments that minimizes (or maximizes) the overall cost of the assignments. A mathematical description of the optimization problem is to find a minimum (or maximum) weight matching in a complete weighted bipartite graph. Given two disjoint sets of arbitrary objects *X *= (*x*_1_, *x*_2_,..., *x*_*n*_) and *Y *= (*y*_1_, *y*_2_,..., *y*_*m*_) with *n *≥ *m *without loss of generality, the optimization problem can be defined as:(1)

where *π *is a subset of the indices 1,..., *n *of size *m *describing the assigned objects of the set *X *and *w*(*x*_*π*(*i*)_, *y*_*i*_) is the weight (cost) of mapping the object *x*_*π*(*i*) _onto *y*_*i*_. The Kuhn-Munkres algorithm (also called Hungarian Method) [42,43] is one popular algorithm to solve this optimization problem.

Fröhlich et al. introduced the concept of the optimal assignment in the field of cheminformatics as a kernel function for attributed molecular graphs [29,30]. Although this function is not a valid kernel function [44], it can be used as a similarity function based on molecular graphs.

### Optimal Assignment Kernel

The idea of the Optimal Assignment Kernel (OAK) [29,30] is to find a mapping of the atoms of the smaller molecule onto the atoms of the other molecule, maximizing the sum of the pairwise atom similarities. The first step of this approach is the calculation of all pairwise atom similarities. For this purpose, a radial basis function (RBF) calculates a similarity value based on physico-chemical descriptors of the atoms (Equation 2, where *a*_*i *_and *b*_*i *_are the *i*th descriptor value of the atom *a *and *b*, *σ*^2 ^represents the variance of a descriptor). To calculate the similarity between two atoms, the OAK uses 24 atom and 8 bond descriptors of the expert system of JOELib2 [45,46]. For a complete list of the descriptors, we refer to Additional file 1 of this study.(2)

To improve the chemical interpretation of the similarity calculation, the information of the topological neighbors and the bonds up to a predefined depth is integrated into the atom-wise similarity calculation. The integration is realized by a recursive atom-wise similarity calculation of the neighbors. In addition, a decay parameter reduces the influence of atoms with increased topological distance. Given two molecules *A *and *B *with atoms *a*_1_,..., *a*_*n *_and *b*_1_,..., *b*_*m *_the primal form of the optimal assignment problem is modified to Equation 3.(3)

The result of Equation 3 is the sum of all atom-wise similarity values. Hence, the function yields higher results if the molecules have more atoms. To reduce the impact of the number of atoms on the final similarity value, the result of the optimal assignment is normalized by Equation 4 to a final value in the range of [0; 1].(4)

**Figure 1:**
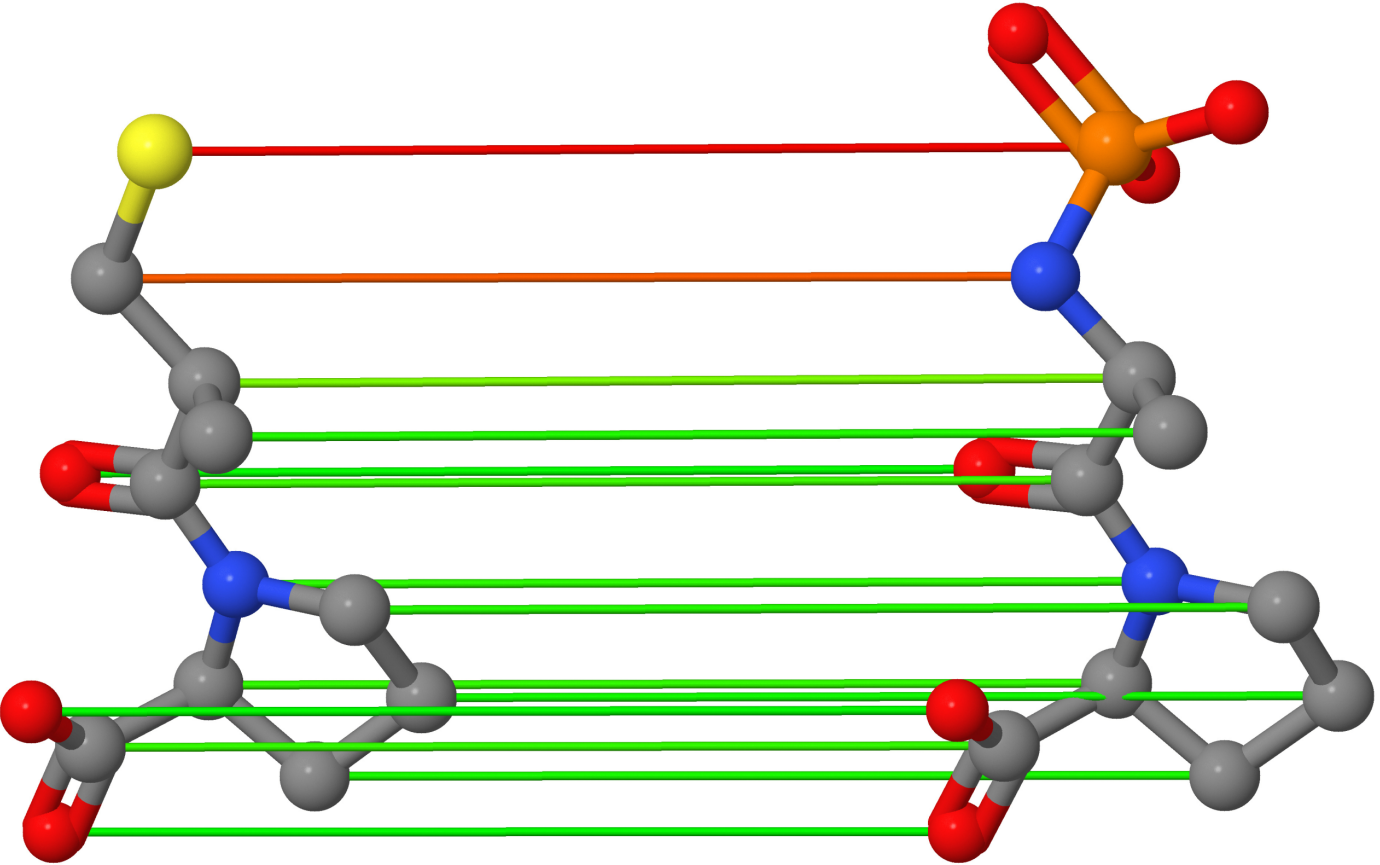
**Optimal atom assignment**. Optimal atom assignment of two angiotensine-converting enzyme molecules. The assignments are based on local atom similarity calculations of the OAK. The color of the mapping edges indicates the atom similarity: green represents a high similarity whereas red edges indicate a low similarity.

An example of an optimal assignment on molecular graphs can be seen in Figure 1.

### Optimal Assignment Kernel with Flexibility Extension

**Figure 2:**
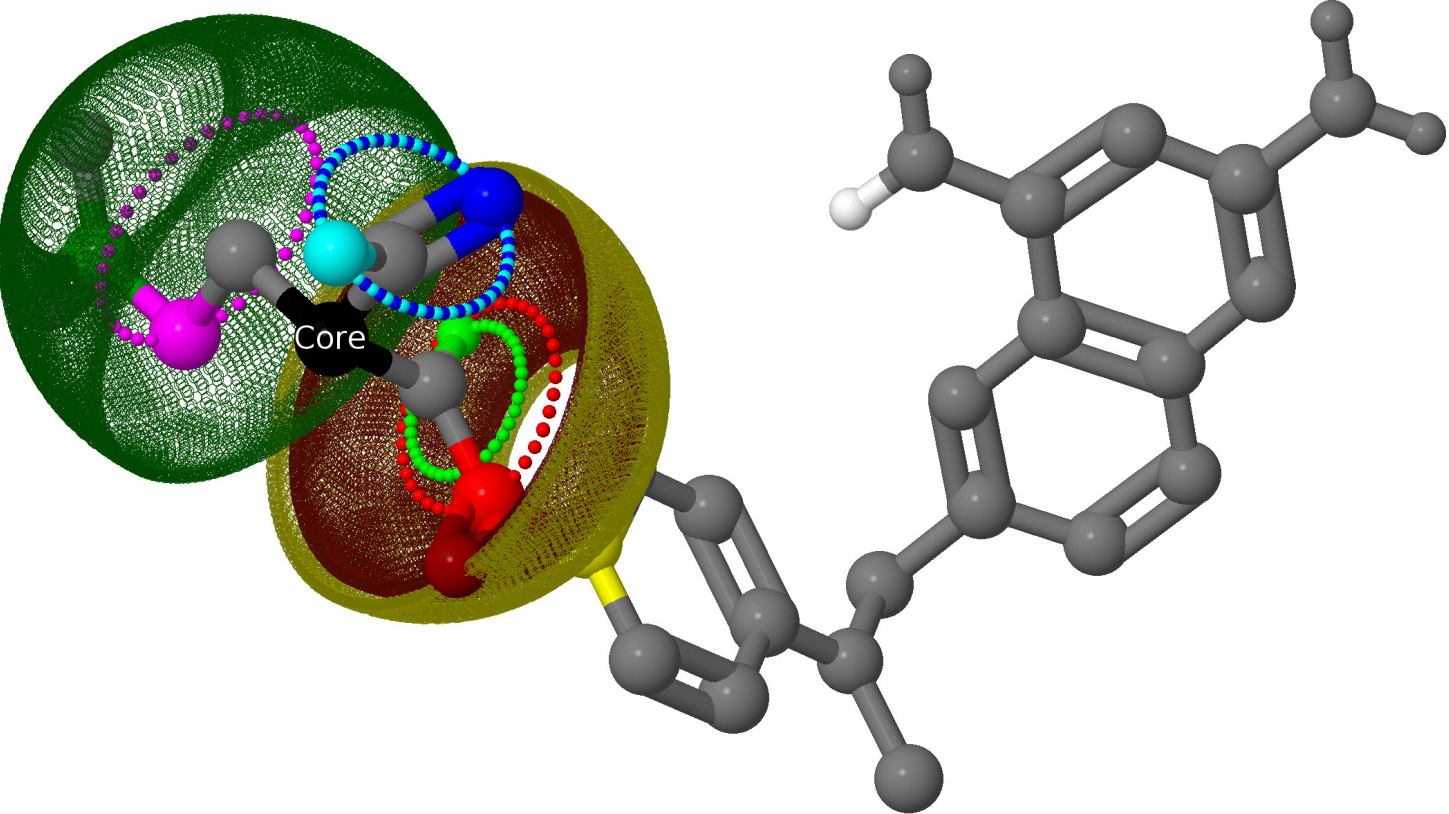
**Local flexibility**. Visualization of the local flexibility for one core atom. The colored shapes represent possible positions of the equal colored atoms. The black core atom is the source of the flexibility objects.

The original OAK computes the similarity of two molecules using the sets of atoms augmented with their local neighborhood. The idea of the OAK can also be applied to approximate the similarity of the conformational space of two molecules [34]. This is achieved by breaking down the overall molecular flexibility into a set of local flexibilities similar to the local atom environments used by the OAK. The local flexibility is defined as the spatial positions on which the neighbors of a center (core) atom could be found depending on the length and angles of the bonds by which they are connected to the core (e.g. Figure 2). Only the flexibility of the second and third order neighbors are considered and the information is stored in the core atom.

The spatial positioning possibilities are expressed by using internal coordinate parameters like bond lengths and angles. These parameters are regarded as only depending on the hybridization of the atoms that connect the core with the neighbor. Non-bonding interactions like electrostatic and steric interactions, which would also influence the conformational space, are not considered. Ring bonds and non-single bonds are generally regarded as rigid. Therefore, the approach only approximates the real spatial positioning of the neighbor. The details of the parametrization of the local flexibility can be found in [34].

The overall similarity of the approximated conformational spaces is computed by augmenting the local atom similarity in the original OAK formulation with the similarity of the local flexibility (i.e. the parametrization of the spatial positioning of the neighbors) of the center atoms (OAK_FLEX_). The flexibility similarity between two atoms is the weighted sum of the flexibility similarities of the second and third order neighbors. The weighted sum is adjusted by an internal parameter. Additionally, the contribution of the flexibility similarity to the original OAK similarity is set by a second parameter. We used the first default parametrization as suggested by Fechner et al. [34] (OAK weight = 0.95, flexibility extension weight = 0.05, second order neighbor weight = 0.3, and third order neighbor weight = 0.7)

### Two-Step Hierarchical Assignment

**Figure 3:**
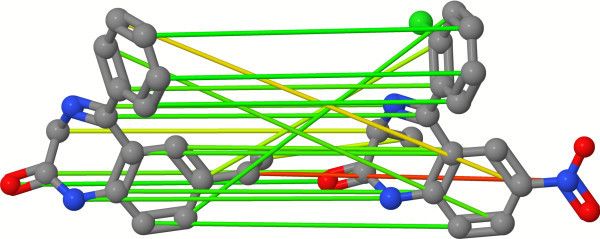
**Optimal atom assignment with topological errors**. Atom mapping of the OAK on two benzodiazepine derivatives disclosing topological errors. Each of the four intersecting edges maps one atom of the aromatic system on the condensed system.

An accurate investigation of the assignments of the OAK discloses failures and shows that the OAK is not able to generate a substructure preserving assignment of the atoms in some cases. These topological errors result in an assignment that scatters the atoms of a substructure over the complete other molecule. The assignments maximize the overall similarity, but from a chemical point of view some mappings are problematic. An example of an OAK assignment with topological errors can be seen in Figure 3.

**Figure 4:**
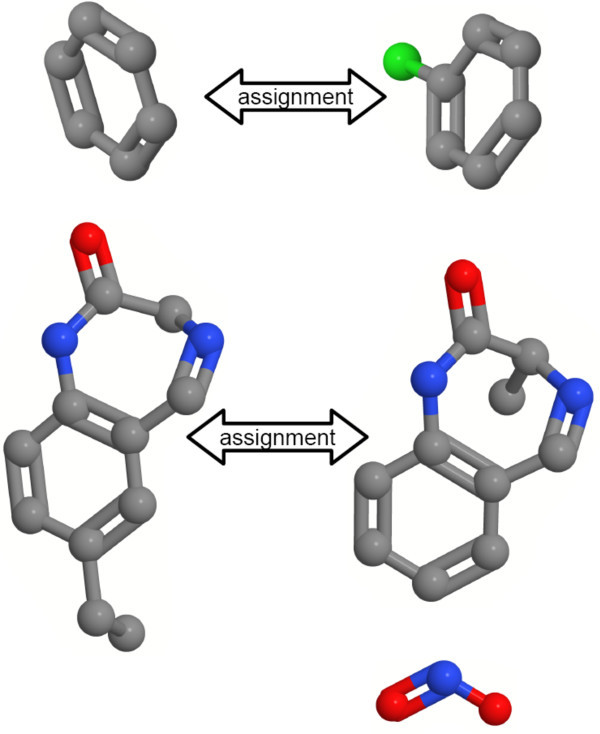
**Fragmentation and assignment of fragments**. Result of the fragmentation algorithm and the first assignment step. The aromatic and condensed systems were mapped onto each other. The terminal nitro group forms a conjugated fragment but has no assignment partner and remains unassigned.

Our analysis uncovered that the occurrence of these errors is favored by the existence of multiple aromatic or condensed ring systems with a small number of substitutions and the presence of conjugated environments. To reduce the number of topological errors, we developed a two-step hierarchical assignment approach (2SHA). The basic idea is to perform two different assignments on different types of objects. The first assignment step operates on a substructure level and maps similar substructures of the molecules onto each other. For this purpose, a preprocessing step is necessary to identify condensed, aromatic, and conjugated systems (e.g. Figure 4). The ring detection is done with a modified biconnected component algorithm that operates on the molecular graph. The identification of the aromaticity and conjugated environments is done by the expert system of JOELib2 [45,46]. To compute the similarity between two substructures, we use a modified version of the original OAK that computes a similarity score without performing the normalization as given in Equation 4. The advantage of omitting this step is a reduced computation time because the self-similarities Sim(*A, A*) and Sim(*B, B*) are not necessary and are not computed.

The algorithm computes the first optimal assignment calculation based on the substructure similarity values and maps one fragment of the smaller molecule on exactly one fragment of the other molecule. An example of such a mapping process is shown in Figure 4 on the two benzodiazepine derivatives used to explain the topological errors. The information of the fragment-based mapping is stored in each atom of a mapped fragment. This knowledge is used to establish constraints for the second assignment on the atomic level that reduces the frequency of topological errors.

An additional aim of this approach is the inclusion of geometrical information into the similarity calculation. To achieve this, we integrate an extra calculation step between the two assignment calculations. The initial preprocessing of the structures identifies aromatic systems, condensed rings, and conjugated environments, which are all rigid scaffolds of molecules. Our idea is to superimpose the rigid substructures which were mapped in the first assignment step. To realize the superposition, the substructures are treated as individual molecules and an algorithm based on quaternions, which represents an equivalent method to the well-known Kabsch algorithm, was used [47-49]. Note that the necessary information of matching atoms of the Kabsch algorithm is the result of the similarity calculation between the substructures. The reason for this is the optimal assignment of the atoms of the substructures to calculate the similarity. Although the algorithm calculates a superposition minimizing the root-mean-square deviation, our approach uses the three-dimensional atom coordinates and integrates the information into the second assignment step.

The second assignment is the final optimal assignment on the atomic level, which includes all information of the previous steps. Each atom has information about its integration into substructures and the mapping of these substructures. Considering this information, all atoms of the molecules can be divided into three different atom classes: First, atoms that are not part of a substructure, like linkers or side-chains. Second, atoms that are part of fragments which were not mapped in the first assignment. Third, atoms that are part of fragments which were mapped. Using these three classes we obtain six different unordered 2-element subsets. Each subset represents a different case of the atom-wise similarity calculation using different information and parameters. The case in which both atoms are part of a fragment and both fragments were not mapped cannot occur because of the nature of the optimal assignment problem. This reduces the number of different cases to five, which have to be regarded in the similarity calculation on the atomic level. However, the case in which both atoms are part of a fragment has to be divided into two cases, depending on whether the fragments were mapped onto each other or not. So we need to consider six cases overall. To include this discrimination, we integrated the following case differentiation into the original atom-wise similarity calculation:

1. Both atoms are not part of a fragment. Calculate the similarity using the original RBF of the OAK.

2. Both atoms are part of a fragment and the fragments were not mapped onto each other. This case is the main cause of topological errors. Therefore, the method has to penalize the similarity score to avoid a mapping of those atoms. The penalization is done by a decreased *σ *value in the RBF. This modification sharpens the RBF and reduces the overall score of the atom-wise similarity calculation.

3. Both atoms are part of a fragment and the fragments were mapped onto each other. The method makes use of the geometrical information of the superposition of the fragments. Given two atoms from the two superimposed fragments, the method calculates a vector based on the two atom coordinates and maps the vector into a two-dimensional plane using an isometric mapping. The initial and terminal points of the two-dimensional vector represent the coordinates of the atoms in the two-dimensional plane. An RBF is centered at each point using the van der Waals radius of the corresponding atom as *σ *value (*σ*_vdW_). Equation 5 calculates the final geometrical similarity value for two mapped atoms *a *and *b *using two RBF and the half atomic distance  as input. The result of this geometrical atom-wise similarity calculation is integrated into the original RBF of the OAK in the form of an additional numerical descriptor.(5)

4. One atom is part of a fragment, but this fragment is not mapped. The other atom is not part of a fragment. In this case the method tries to map an atom of a rigid fragment onto a side-chain or linker atom. The fragment was not mapped in the first assignment step, but this mapping is also doubtful and has to be penalized. The technique for penalizing those mappings is the same as in the second case.

5. One atom is part of a fragment which is mapped. The other atom is not part of a fragment. This case is related to the previous one, but the fragment was mapped in the first assignment step. If the atom-wise similarity calculation results in a high similarity value, the possibility that the atom of the fragment is assigned to an atom of the corresponding fragment of the other molecule is reduced. Depending on the structure of the two molecules, this increases the possibility of topological errors. Therefore, the penalty has to be higher than in the fourth case.

6. Both atoms are part of a fragment, but only one fragment was mapped. This case is also related to the fourth and fifth one, but the mapping of two atoms of fragments is more reasonable from a chemical point of view. However, the risk of topological errors is also increased, and therefore we use the same penalization as in the fourth case.

The separate *σ *values of each case can be set individually and have a great impact on the final similarity value. The optimal parametrization depends on the structures of a data set. Based on a chemical interpretation of the different cases, we expected the following relations between the different *σ *values: *σ*_2 _<*σ*_5 _<*σ*_6 _≤ *σ*_4_, where the indices represent the number of the case. We performed a grid search in the range [0.25, 0.5, ⋯, 10.0] to define the *σ *values for the penalization and to validate our hypothesis concerning the relation between the values. The result of the grid search yields the following values: *σ*_2 _= 2.5, *σ*_5 _= 5.0, *σ*_6 _= 4.0, and *σ*_4 _= 4.0. The empirical results of the grid search differ only in the relation between the *σ *values of the fifth and sixth case. Thus, the different cases of the 2SHA method show a correlation with the chemical interpretation.

**Figure 5:**
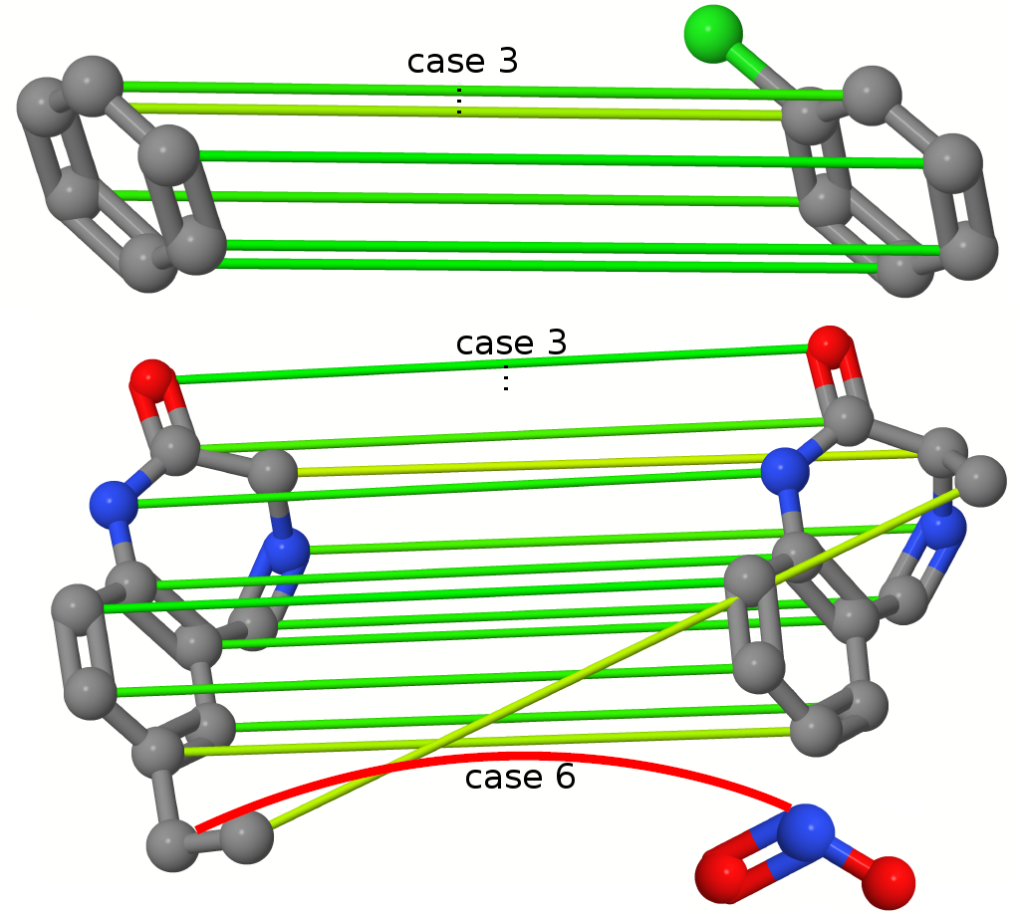
**Optimal atom assignment using the two-step hierarchical assignment**. Result of an optimal assignment using the two-step hierarchical assignment approach with the case differentiation of the pairwise atom similarity calculation. The hierarchical assignment reduces the number of topological errors and shows a substructure preserving mapping. The mapping of the carbon atom of the condensed ring system onto the nitrogen of the nitro group is an example of the sixth case and results in a penalized mapping.

The final result of the optimal assignment of the atoms, based on the atom-wise similarity calculations using the differentiation with the six cases, is shown in Figure 5 on the same molecules causing topological errors using the original OAK. The assignment preserves the mapping of the substructures and reduces the occurrence of topological errors. The assignment of the atom from the condensed ring system onto the nitrogen of the nitro group is penalized.

### Optimal Local Atom Pair Environment Assignment

This variant of the optimal assignment approach uses local atom pair environments (OAAP). There are two fundamental types of atom pairs: topological and geometrical atom pairs. We employed the binned geometrical distance matrix  between the three-dimensional coordinates of atoms *i*, *j *of a molecule in this study. The geometrical bin size *b *was set to 1 Å. Therefore, this method can be considered as a geometrical similarity measure. The computation time of *D *has a quadratic complexity with the number of atoms. The matrix is symmetric, so it is sufficient to compute the upper half of each matrix. The distance in the diagonal equals zero.

**Figure 6:**
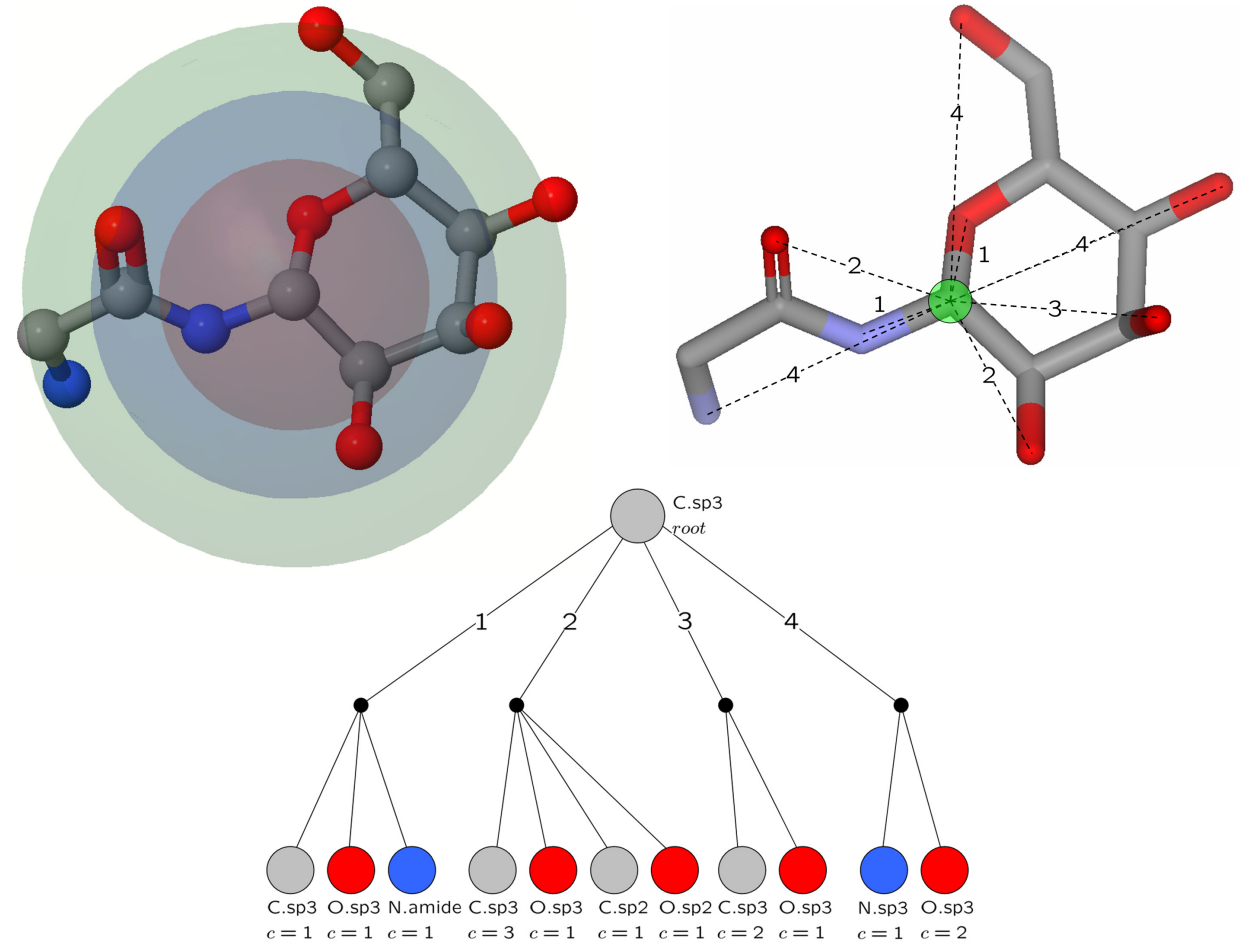
**Binned geometrical distances, spheres and trie**. The upper left figure shows the spheres of the binned geometrical distances of 1.0, 2.0, and 3.0 Å for the centered carbon atom. The sphere of the binned geometrical distance of 0.0 Å (distances in the range [0.0;1.0)) is not visualized as individual sphere because it contains no atoms. The upper right figure illustrates the resulting local atom pair environment of binned geometrical distances. For simplicity, only the distances to non-carbon atoms are displayed. The lower figure visualizes the corresponding trie of geometric atomic distances of the annotated atom in the upper figures. The root and leaves are labeled with the corresponding atom type. The leaves contain additionally the total number of occurrences in the local atom pair environment.

*D *is used as lookup table to store the information of all geometrical atom pairs from atom *i *in a trie. A trie is a prefix search tree that can be applied to patterns with a reading direction. In our case, we have a star-shaped local atom environment. At the root of the trie of atom *i *the hash code of the atomic symbol *i *is placed. Next, the patterns of the form

are inserted as ordered triplets. Note that *symbol *can be any atom type or fragment representation and *hash*(*symbol*) any suitable hash function *hash *: *symbol *→ ℕ. By using a trie, the collection of patterns is non-redundant, because the trie is updated whenever a known part of a pattern is inserted. If the whole pattern is already contained in the trie, the count is incremented by one. For many similarity metrics, it is necessary to know basic properties of a trie, for example the total number of patterns or the number of unique patterns (number of leaves). Therefore, our trie implementation has a method to compute such properties. A geometrical local atom pair environment and the corresponding trie of the marked atom is shown in Figure 6.

The representation of the local atom environments as tries allows us to apply efficient recursive similarity computations. A well-known similarity metric for nominal features is the Tanimoto coefficient. The computation of the Tanimoto coefficient of two local atom environments is reduced to a comparison of two tries.

Let *L*_*A*_, *L*_*B *_be the sets of local atom pair environments of two molecular graphs *A, B *and  the tries *i*, *j *of the nominal features (atom pair environments of atoms *i*, *j*). Now, the Tanimoto coefficient can be defined as:(6)

The overall molecular similarity is computed by the score of the optimal assignment of the local atom pair environments. The cost of computing the local similarity matrix is *O*(*nml*) for *n*, *m *atoms of the molecules *A, B *and *l *leaves of the larger trie. The lookup for a pattern has a constant computation time, because the depth of the tries is fixed.

We computed the OAAP similarity between two structures on single low-energy conformations. In spite that, it is possible to extend this method to conformational ensembles. This could be accomplished by simply averaging the distances as a case in point. The approach was implemented using the Chemistry Development Kit (CDK) library [50,51].

### Parameters and Computation Time

All presented methods have internal parameters, which allow modifications of the similarity measures. These modifications can improve the results on a specific problem and data set. The optimization of the parameters is a complex process making extensive methods like grid searches necessary. However, one aim of this work was to evaluate the usability of optimal assignment methods on molecular graphs for ligand-based VS experiments. Therefore, we used the described standard parameters for each method, which were constant for all data sets to obtain comparable results of the overall performance.

The computation time of the methods depends on the number of atoms, which have to be mapped in the assignment step. In addition, each method has its own type of preprocessing and mining the chemical information. This yields differences in the performance depending on the data set. Instead of giving a complete overview of the computation time for each method and data set, we report an averaged computation time, as it can be expected for drug-like structures, for each method. The original OAK has an averaged performance of 27.34 ± 3.40 similarity calculations per second. The flexibility extension OAK_FLEX _yields 41.03 ± 7.32 calculations per second. This speed-up of the OAK_FLEX _is the result of a reduction of the used features. The OAAP method achieves a performance of 51.49 ± 18.07 similarity calculations per second (computation time for the atom typing is not included). The 2SHA method has the highest computation time, because of the two assignment steps and the superposition of the fragments. However, the approach is capable to perform 14.04 ± 1.78 calculations per second. All values were measured on a Intel Core2Duo CPU with 2 GHz using one core and 1 GB memory, Java 1.6.0_07, Ubuntu 8.04.3, Linux kernel 2.6.24.

### Experimental

The setup of the VS experiments is based on the work of Cheeseright et al. in which the FieldScreen approach is introduced and compared against a docking algorithm. We decided to use this workflow to create a common setup of the data sets with the objective to achieve comparable results.

### Data Sets and Preparation

All ligand-based VS experiments were performed using a modified version of the Directory of Useful Decoys (DUD) Release 2 [52,53]. The DUD contains known actives and *mimetic *[39] decoys for 40 target proteins. This collection of data sets was compiled to serve as an unbiased community benchmark database for the evaluation of docking algorithms. Therefore, the original version of the DUD is not suited for ligand-based VS experiments [41]. A modification of the active structures of the DUD database, suggested by Good and Oprea [35], solves this problem by applying a lead-like filter [54] and a cluster algorithm [55]. The clustering identifies analogue structures and can be used to reduce the bias of analogue or trivial enrichment. Additionally, each cluster can be seen as an individual chemotype and allows the analysis of the "scaffold-hopping" behavior of the methods.

The active structures for the VS experiments were prepared as follows. We obtained the modified active structures of Good and Oprea from the DUD site [38]. These data sets contain only topological information about the structures. Thus, we used CORINA3D [56] to generate initial geometrical seed structures. Those were refined with MacroModel 9.6 [57] using the OPLS 2005 force field and the limited Broyden-Fletcher-Goldfarb-Shanno optimization method with 5000 iterations and a gradient criterion of 0.0001 RMSD for the atomic movement between two iterations. The final data sets were used as active structures.

The original decoys of the DUD release 2 were obtained from the DUD site [52]. Using these decoys, together with the filtered actives from Good and Oprea, would introduce a bias of an artificial enrichment based on the distinction of physical property values. To remove this artificial enrichment bias, we applied the same lead-like filter, used by Good and Oprea to filter the active structures, on the decoys. Thus, the AlogP value and the molecular weight was calculated for each structure with dragonX 1.4 [58]. All structures with an AlogP value *> *= 4.5 (5.5 for nuclear hormone receptor data sets) or a molecular weight  were removed. The initial three-dimensional coordinates were further optimized using the same configuration of MacroModel as for the active structures.

The described preparation protocol was applied on all 40 data sets of the DUD. The SD files of the prepared actives and decoys are directly obtainable from the DUD site [52].

To obtain results that are not biased by a low number of chemotypes, it is advisable to use data sets with a sufficient number of chemotypes. Therefore, we used the same subset of DUD targets as used by Cheeseright et al. This subset has a minimum of 17 and a maximum of 44 chemotypes. A comprehensive overview of the data sets can be seen in Table 1.

**Table 1:** Data sets.

target	number actives	number decoys	number clusters^a^	PDB code^b^
ace^c^	46	1796	19^p^	1o86
ache^d^	100	3859	18	1eve
cdk2^e^	47	2070	32	1ckp
cox2^f^	212	12606	44	1cx2
egfr^g^	365	15560	40	1m17
fxa^h^	64	2092	19	1f0r
hivrt^i^	34	1494	17	1rt1
inha^j^	57	2707	23	1p44
p38^k^	137	6779	20	1kv2
pde5^l^	26	1698	22	1xp0
pdgfrb^m^	124	5603	22	1t46
src^n^	98	5679	21	2src
vegfr2^o^	48	2712	31	1fgi

Overview of the used data sets containing the number of actives, decoys, different chemotype clusters, and the PDB code of the complexed crystal structure which contains the search query.

^a^Number of clusters using the reduced graph algorithm from Barker et al. [55]

^b ^PDB code of the complexed crystal structures from which the search queries were taken.

^c ^Angiotensine-converting enzyme

^d ^Acetylcholinesterase

^e ^Cyclin-dependent kinase

^f ^Cyclooxygenase-2

^g ^Epidermal growth factor receptor

^h ^Factor Xa

^i ^HIV reverse transcriptase

^j ^Enoyl ACP reductase

^k ^P38 mitogen activated protein

^l ^Phosphodiesterase 5

^m ^Platelet derived growth factor receptor kinase

^n ^Tyrosine kinase

^o ^Vascular endothelial growth factor receptor

^p ^The molecule with the ZINC ID 03814157 does not contain a ring and therefore it is assigned to a dummy cluster forming one additional cluster.

Ligand-based VS methods need a biologically active structure that serves as a query in the experiment. In the work of Huang et al. [53] complexed crystal structures were used to identify the binding sites for the docking algorithm. Cheeseright et al. [27] used the ligands of the same complexed crystal structures as query structures to evaluate the FieldScreen approach. To allow a comparison with FieldScreen, we extracted the same bounded ligands from the protein data bank [59,60] and corrected the bonds lengths with MacroModel 9.6. These structures serve as queries for the VS experiments in this study.

### Evaluation of VS Performance

In the following section we describe the metrics for the evaluation of VS experiments used in our work. Additionally, we explain why we used these metrics and show their correlation to well-known established metrics.

The evaluation and comparison of VS performance is an important but also an error-prone process [39,40,61]. In recent years, a plethora of evaluation metrics have been published, each metric with its strengths and weaknesses. For an overview, we refer to the work of Kirchmair et al. [62] There is no standard protocol for the analysis and publication of VS results. Thus, the comparison of different works is a challenging task. It is necessary to analyze the results, regarding three different aspects, to characterize the performance of a VS method. The first aspect is the early recognition problem and originates from real-world screening applications, where only top ranked molecules were selected for testing in biological assays because of high costs and expenditure of time. Truchon and Bayly developed for this purpose the BEDROC score [63], which uses a decreasing exponential weighting function. The drawback of this metric is its lack of interpretability and the dependency of an extrinsic variable [39]. To resolve these problems, Jain and Nicholls suggested to report the enrichment values at predefined false positive fractions [40]. These values can be obtained by dividing the sensitivity value by the fraction of false positives. Equation 7 represents the interrelation between the ROC value and the ROC enrichment for a predefined false positive fraction.  and  is the number of true positives (actives retrieved) and false positives (decoys retrieved), respectively, found in the range containing a false positive rate of *X*%. TP, TN, FP, and FN are the entries of the confusion matrix ranking *X*% false positives.(7)

We decided to report the ROC enrichments at false positive rates of 0.5%, 1.0%, 2%, and 5%, as suggested by Jain and Nicholls [40].

The second aspect of characterizing the performance of VS methods is the evaluation of the performance considering the complete data set. This overall performance can be visualized plotting the receiver operating characteristic (ROC), which should always be part of VS results [39,64]. However, a comparison of many ROC plots is not straightforward, thus we report the area under the ROC curve to provide a better overview of the results. Equation 8 shows the calculation of the area under the ROC curve (AUC), where *N*_actives _and *N*_decoys _are the numbers of active and decoy structures, respectively  is the number of decoys that are higher ranked than the *i*th active structure.(8)

The last point of characterizing the performance of VS methods is the evaluation of the retrieval of new scaffolds. As already mentioned, ligand-based VS suffers from the bias caused by enrichments of analog structures. To reduce the influence of this overestimated enrichments on the result metrics, Clark and Webster-Clark recommended an adaption of the ROC and AUC calculation [36]. They proposed an arithmetic and a harmonic weighting scheme to reduce the influence of structurally similar structures. Using the arithmetic weighting, each structure has a weight that is inversely proportional to the size of the cluster it belongs to. Therefore, the weight of all structures taken from one cluster is equal. This leads to the equation: , where *w*_*ij *_is the weight of the *i*th structure taken from the *j*th cluster with *N*_*j *_structures. As a result of this, the true positive value of the sensitivity is no more the number of true active structures seen in a predefined fraction of the data set. Instead, it is the sum of the weights resulting in the equation  The value of  is 1 if the *i*th structure of the *j*th cluster is contained in the fraction of the data set, otherwise it is 0. *N*_*clusters *_is the number of clusters in the data set and *N*_*j *_is the number of structures in the *j*th cluster.

The harmonic weighting scheme uses weights representing the ranks of the active structures within each cluster. Top-ranked structures of a cluster have a higher weight, motivated by the idea that those structures have the highest information content. The weight of the structures is defined as , where *i *is the *i*th structure of the *j*th cluster in decreasing rank order. A recently published analysis of the weighting schemes conducted by Mackey and Melville discloses that the arithmetic weighting scheme has better properties and is more robust than the harmonic scheme [65]. Therefore, we decided to use the arithmetic weighting scheme. Integrating this scheme into the basic ROC enrichment leads to an arithmetic weighted version (awROC enrichment) given by Equation 9.(9)

This weighting scheme can also be embedded into the AUC calculation. The modified version (awAUC) can be seen in Equation 10.(10)

To account for intrinsic variances [39] of the active and decoy structures, we bootstrapped the data sets and approximated the error of the awROC enrichments and awAUC. We performed 25000 iterations removing randomly 20% of the data set. The standard deviation of the 25000 metric results were used as an error of the result using the complete data set.

### Comparison against Other Methods

The docking study by Huang et al. [53] provides the energy scores of the DOCK algorithm for each target of the DUD. We used these rankings to calculate the awROC enrichments and awAUC on the same data sets as our approaches. Hence, extensive properties such as active to decoy ratio and data set size are identical and permit a comparison of the results. A direct comparison of docking algorithms with ligand-based VS tools is critical because of the different techniques and information included in the methods. We calculated the results of the docking algorithm with the objective to provide an impression of the performance of the DOCK algorithm using the awROC and awAUC evaluation metrics.

We considered the results of the FieldScreen approach to include a sophisticated ligand-based method. To reduce the information content solely to the information obtained from the query structure, the results without the excluded volume of the protein were used. Although the data set sizes and active to decoy ratios of the FieldScreen results deviate from our setup, the use of awROC enrichments ensures the comparability because the ROC enrichment is independent from extensive quantities [39].

Introducing a new method to a well-established field, like ligand-based VS, it is necessary to justify it by comparing the results to a common approach. To meet these requirements, we compared our approaches to the 166 bit MACCS keys [12] using the Tanimoto coefficient to calculate a similarity value. Although the results of the MACCS keys are inferior in comparison to other fingerprints [66] we provide the results of the MACCS keys to give a baseline for the performance on the data sets. The MACCS keys were calculated using the CDK 1.1.5 [50,51].

## Results and Discussion

The results of the four optimal assignment based methods and the three comparison approaches are organized as follows. The awROC enrichments for each target at a predefined false positive fraction of 0.5%, 1.0%, 2.0%, and 5.0% can be seen in the Tables 2, 3, 4 and 5. The awAUC values are compiled in Table 6. Each value in the Tables 2, 3, 4, 5 and 6 has a standard deviation, which is the result of the error approximation using the bootstrapping approach. The bold value in each row indicates the best result concerning the data set and evaluation metric. The last row in each Table contains the rank of each method averaged over all data sets. These values describe the overall performance of one method considering the results of all other methods.

**Table 2:** awROC Enrichments at 0.5%.

target	DOCK	FieldScreen	MACCS	OAK	OAK_FLEX_	2SHA	OAAP
ace	17.0 ± 6.2	14.7	55.7 ± 8.5	**94.5 **± **8.7**	94.5 ± 9.6	73.5 ± 8.6	30.5 ± 8.4
ache	0.0 ± 0.0	16.7	19.1 ± 4.6	24.5 ± 4.8	**24.9 **± **4.8**	24.8 ± 4.9	23.1 ± 4.7
cdk2	4.0 ± 6.1	7.5	9.4 ± 1.8	9.4 ± 1.8	9.4 ± 1.8	9.4 ± 3.7	**24.1 **± **4.7**
cox2	1.9 ± 0.6	48.8	17.0 ± 3.0	25.7 ± 3.6	42.5 ± 4.5	38.3 ± 6.4	**68.4 **± **5.5**
egfr	7.6 ± 2.2	52.4	40.3 ± 3.2	56.6 ± 3.9	47.4 ± 3.6	**103.6 **± **6.9**	53.1 ± 4.3
fxa	15.1 ± 6.8	0.0	**30.0 **± **7.4**	20.0 ± 6.3	10.0 ± 4.6	10.0 ± 4.6	20.0 ± 6.3
hivrt	4.4 ± 1.8	**40.0**	22.0 ± 5.5	20.1 ± 5.6	20.1 ± 5.6	20.1 ± 5.6	34.8 ± 8.0
inha	0.0 ± 0.0	56.7	49.9 ± 7.7	31.8 ± 8.7	43.9 ± 8.4	57.9 ± 13.5	**60.7 **± **7.4**
p38	0.0 ± 0.0	3.7	1.0 ± 0.4	22.2 ± 5.9	18.8 ± 5.0	**28.2 **± **6.3**	10.7 ± 4.3
pde5	4.4 ± 4.3	6.8	4.3 ± 3.3	**8.6 **± **4.0**	**8.6 **± **4.0**	4.3 ± 2.3	0.0 ± 0.0
pdgfrb	0.0 ± 0.0	27.3	42.0 ± 7.4	47.0 ± 6.7	44.4 ± 6.7	43.9 ± 6.7	**57.7 **± **6.8**
src	0.0 ± 0.0	**13.7**	0.0 ± 0.0	5.7 ± 1.2	9.2 ± 1.2	4.3 ± 1.6	4.7 ± 1.0
vegfr2	6.2 ± 3.0	12.9	**18.7 **± **4.5**	6.3 ± 1.6	12.5 ± 3.4	12.5 ± 3.4	12.5 ± 4.1

avg. rank	6.27	4.08	4.35	3.23	3.27	3.54	3.12

awROC Enrichments at 0.5% false positive fraction of DOCK, FieldScreen, MACCS keys, and the optimal assignment methods.

**Table 3:** awROC Enrichments at 1.0%.

target	DOCK	FieldScreen	MACCS	OAK	OAK_FLEX_	2SHA	OAAP
ace	13.5 ± 4.1	12.6	36.8 ± 4.9	47.3 ± 4.8	**52.5 **± **4.8**	42.0 ± 4.9	20.5 ± 4.3
ache	0.0 ± 0.0	**20.4**	9.8 ± 2.4	13.0 ± 2.5	14.2 ± 2.6	15.2 ± 2.8	11.8 ± 2.4
cdk2	9.5 ± 2.7	3.8	4.9 ± 1.0	6.5 ± 1.5	4.9 ± 1.5	11.1 ± 2.4	**15.7 **± **2.8**
cox2	5.3 ± 1.4	29.5	10.7 ± 1.7	17.2 ± 2.0	24.0 ± 2.4	33.7 ± 2.9	**46.1 **± **3.1**
egfr	7.7 ± 1.6	29.5	20.4 ± 1.6	40.3 ± 3.1	26.2 ± 1.8	**56.2 **± **2.5**	28.3 ± 2.0
fxa	13.5 ± 3.6	2.8	**15.7 **± **3.9**	10.5 ± 3.3	5.2 ± 2.4	5.2 ± 2.4	10.5 ± 3.3
hivrt	2.2 ± 0.9	11.7	10.7 ± 3.7	10.7 ± 2.8	10.7 ± 2.8	10.7 ± 2.8	**19.6 **± **4.0**
inha	0.0 ± 0.0	31.2	31.0 ± 4.0	25.2 ± 3.6	22.9 ± 3.6	**42.3 **± **4.2**	31.8 ± 3.7
p38	0.0 ± 0.0	1.8	0.5 ± 0.2	14.1 ± 3.2	11.8 ± 2.5	**16.3 **± **3.2**	5.6 ± 2.2
pde5	**6.7 **± **3.1**	4.5	2.3 ± 1.7	4.5 ± 2.0	4.5 ± 2.0	2.3 ± 1.2	0.0 ± 0.0
pdgfrb	0.0 ± 0.0	13.6	23.2 ± 3.5	23.9 ± 3.4	22.9 ± 3.4	22.3 ± 3.4	**31.4 **± **3.4**
src	4.7 ± 2.1	7.0	0.0 ± 0.0	3.8 ± 0.5	5.4 ± 0.6	**9.7 **± **2.2**	2.8 ± 0.5
vegfr2	3.2 ± 1.5	8.1	**9.4 **± **2.2**	3.1 ± 0.8	6.3 ± 1.7	9.4 ± 3.1	**9.4 **± **2.2**

avg. rank	5.54	4.00	4.73	3.65	4.04	2.73	3.23

awROC Enrichments at 1.0% false positive fraction of DOCK, FieldScreen, MACCS keys, and the optimal assignment methods.

**Table 4:** awROC Enrichments at 2.0%.

target	DOCK	FieldScreen	MACCS	OAK	OAK_FLEX_	2SHA	OAAP
ace	8.2 ± 1.9	8.9	21.3 ± 2.4	**30.2 **± **2.6**	27.6 ± 2.5	21.0 ± 3.0	14.7 ± 2.6
ache	0.0 ± 0.0	**13.5**	5.3 ± 1.2	7.5 ± 1.3	7.5 ± 1.3	10.1 ± 1.4	6.3 ± 1.3
cdk2	7.0 ± 1.4	1.9	2.5 ± 0.5	5.5 ± 1.2	4.8 ± 1.1	7.9 ± 1.4	**8.6 **± **1.3**
cox2	5.9 ± 1.3	17.8	6.8 ± 0.8	14.1 ± 1.6	17.6 ± 1.5	22.9 ± 1.4	**24.6 **± **1.4**
egfr	7.2 ± 0.8	18.1	11.0 ± 0.8	24.1 ± 1.3	20.5 ± 1.3	**29.2 **± **1.2**	15.8 ± 1.1
fxa	6.8 ± 1.8	5.4	**7.9 **± **2.0**	5.3 ± 1.7	2.6 ± 1.2	2.6 ± 1.2	5.3 ± 1.7
hivrt	2.2 ± 0.5	**11.7**	8.8 ± 1.8	8.3 ± 2.1	5.4 ± 1.8	5.4 ± 1.4	9.8 ± 2.0
inha	0.0 ± 0.0	15.6	17.5 ± 2.0	15.9 ± 1.9	13.8 ± 1.7	**22.2 **± **2.1**	16.2 ± 2.0
p38	0.8 ± 0.6	0.9	0.8 ± 0.3	**9.8 **± **1.9**	9.3 ± 1.6	8.8 ± 1.6	5.7 ± 1.7
pde5	7.8 ± 1.7	**9.7**	1.1 ± 0.8	2.3 ± 1.0	3.4 ± 0.8	4.5 ± 0.8	0.0 ± 0.0
pdgfrb	0.0 ± 0.0	9.1	12.8 ± 1.8	12.1 ± 1.7	11.5 ± 1.7	11.3 ± 1.7	**18.8 **± **1.7**
src	2.5 ± 1.1	3.7	0.0 ± 0.0	2.5 ± 0.2	6.4 ± 1.1	**7.7 **± **1.2**	1.6 ± 0.3
vegfr2	3.2 ± 1.1	7.3	6.4 ± 1.3	3.2 ± 0.9	3.2 ± 0.9	**9.5 **± **1.5**	4.8 ± 1.1

avg. rank	6.54	3.62	4.93	3.58	4.12	2.81	3.73

awROC Enrichments at 2.0% false positive fraction of DOCK, FieldScreen, MACCS keys, and the optimal assignment methods.

**Table 5:** awROC Enrichments at 5.0%.

target	DOCK	FieldScreen	MACCS	OAK	OAK_FLEX_	2SHA	OAAP
ace	4.6 ± 0.9	4.7	10.7 ± 1.2	**12.1 **± **1.0**	**12.1 **± **1.0**	11.6 ± 1.0	8.0 ± 1.0
ache	0.8 ± 0.2	**7.3**	2.1 ± 0.5	3.9 ± 0.6	4.4 ± 0.6	5.4 ± 0.6	4.0 ± 0.6
cdk2	2.8 ± 0.6	0.8	2.6 ± 0.6	2.6 ± 0.4	2.6 ± 0.4	**3.5 **± **0.5**	3.5 ± 0.7
cox2	5.5 ± 0.5	10.4	4.9 ± 0.5	9.0 ± 0.6	8.8 ± 0.6	9.7 ± 0.6	**12.2 **± **0.6**
egfr	4.5 ± 0.4	9.5	5.0 ± 0.4	11.6 ± 0.5	11.3 ± 0.5	**12.1 **± **0.5**	7.3 ± 0.5
fxa	**5.5 **± **1.0**	5.4	3.1 ± 0.8	2.1 ± 0.7	1.1 ± 0.5	2.6 ± 0.8	2.1 ± 0.7
hivrt	2.2 ± 0.6	**5.1**	3.5 ± 1.1	3.3 ± 0.8	3.3 ± 0.8	3.5 ± 0.7	**5.1 **± **1.1**
inha	0.0 ± 0.0	6.5	7.0 ± 0.8	8.6 ± 0.8	5.7 ± 0.7	**9.4 **± **0.8**	7.0 ± 0.8
p38	1.4 ± 0.5	0.5	0.5 ± 0.1	4.3 ± 0.7	4.0 ± 0.6	**5.0 **± **0.7**	2.9 ± 0.7
pde5	4.3 ± 0.8	**4.8**	0.5 ± 0.3	2.3 ± 0.6	1.4 ± 0.3	2.7 ± 0.6	1.4 ± 0.6
pdgfrb	0.0 ± 0.0	3.8	6.0 ± 0.7	4.9 ± 0.7	4.9 ± 0.7	4.5 ± 0.7	**8.6 **± **0.7**
src	1.0 ± 0.4	2.5	0.1 ± 0.0	3.7 ± 0.7	4.5 ± 0.8	**6.4 **± **0.7**	1.0 ± 0.1
vegfr2	1.3 ± 0.5	3.5	2.6 ± 0.5	1.3 ± 0.4	1.3 ± 0.4	**4.5 **± **0.7**	2.6 ± 0.5

avg. rank	5.54	3.69	5.04	3.69	4.19	2.23	3.62

awROC Enrichments at 5.0% false positive fraction of DOCK, FieldScreen, MACCS keys, and the optimal assignment methods.

**Table 6:** awAUC values.

target	DOCK	FieldScreen	MACCS	OAK	OAK_FLEX_	2SHA	OAAP
ace	0.67 ± 0.03	0.64	**0.86 **± **0.02**	0.84 ± 0.03	0.81 ± 0.03	0.86 ± 0.03	0.72 ± 0.04
ache	0.57 ± 0.02	**0.62**	0.37 ± 0.03	0.44 ± 0.03	0.46 ± 0.04	0.50 ± 0.04	0.50 ± 0.03
cdk2	0.53 ± 0.03	0.44	**0.55 **± **0.02**	0.55 ± 0.02	0.46 ± 0.02	0.48 ± 0.03	0.53 ± 0.03
cox2	0.68 ± 0.02	0.82	0.56 ± 0.02	0.77 ± 0.02	0.77 ± 0.01	0.79 ± 0.01	**0.87 **± **0.01**
egfr	0.55 ± 0.02	**0.82**	0.60 ± 0.02	0.72 ± 0.02	0.70 ± 0.02	0.72 ± 0.02	0.49 ± 0.02
fxa	0.72 ± 0.03	**0.74**	0.45 ± 0.03	0.46 ± 0.03	0.50 ± 0.03	0.56 ± 0.03	0.57 ± 0.03
hivrt	**0.73 **± 0.02	0.63	0.54 ± 0.04	0.53 ± 0.03	0.47 ± 0.03	0.53 ± 0.04	0.65 ± 0.03
inha	0.26 ± 0.02	**0.72**	0.64 ± 0.03	0.53 ± 0.04	0.52 ± 0.04	0.64 ± 0.03	0.59 ± 0.04
p38	0.36 ± 0.02	0.27	0.38 ± 0.02	0.47 ± 0.03	0.49 ± 0.03	**0.76 **± **0.01**	0.43 ± 0.03
pde5	0.48 ± 0.04	**0.62**	0.28 ± 0.03	0.37 ± 0.04	0.32 ± 0.03	0.38 ± 0.03	0.35 ± 0.03
pdgfrb	0.40 ± 0.02	0.40	0.54 ± 0.03	**0.58 **± **0.03**	0.52 ± 0.03	0.49 ± 0.03	**0.58 **± **0.03**
src	0.52 ± 0.02	0.39	0.50 ± 0.02	0.66 ± 0.02	0.72 ± 0.02	**0.74 **± **0.01**	0.30 ± 0.02
vegfr2	0.42 ± 0.03	0.53	0.42 ± 0.03	0.31 ± 0.03	0.33 ± 0.02	**0.54 **± **0.03**	0.41 ± 0.03

avg. rank	4.27	3.42	4.50	4.04	4.62	3.08	3.92

awAUC values of DOCK, FieldScreen, MACCS keys, and the optimal assignment methods.

In addition to the already mentioned VS metrics, Additional file 2 contains the complete ranking of all structures and several VS metrics for each method and data set.

### Virtual Screening Results

The results of Table 2 show the awROC enrichments at a false positive rate of 0.5%, which implies that the early recognition problem is focused in this evaluation. The best overall performance is achieved by the (OAAP). The results of the original OAK and the flexibility extension are similar and only marginally worse than the OAAP. The 2SHA approach obtains an average rank that lies in between the other optimal assignment methods and FieldScreen. The MACCS keys show an increased performance on the fxa and vegfr2 data set, but the overall performance is lower than all other methods except the DOCK algorithm. The ranking of the methods at higher false positive fractions changes, but there is a tendency observable. The 2SHA yields the best average rank at 1%, 2%, and 5%. Additionally, the average rank decreases with an increasing false positive fraction. FieldScreen shows a similar behavior resulting in comparable results at 5% in comparison to OAK and OAAP. The average ranks of the MACCS keys, OAK, OAK_FLEX_, and OAAP increased with higher false positive fractions by a value of ≈ 0.7. The average rank of the DOCK algorithm alternates between 6.54 and 5.54, showing no dependency on the false positive rate.

The increasing performance of the FieldScreen and 2SHA approach can be explained by the ability to perform "scaffold-hoppings". The other optimal assignment methods have a good early enrichment as seen in Table 2, but these enrichment values are the result of retrievals of chemical similar scaffolds with respect to the search query. Those methods are not able to hold up the high retrieval rates of the chemotypes and the discovery of new chemotypes stagnates. The influence of this bias is reduced with increasing false positive fraction and the more robust methods became apparent. Regarding the technique of FieldScreen, the improved "scaffold-hopping" behavior is probably the result of the conformational sampling together with the similarity calculation which is based on molecular fields. The 2SHA method yields a similar effect by the identification of rigid scaffolds, their assignment and superposition. This procedure can be seen as an inherent heuristic of a flexible alignment of the search query and the screening structures. The method assumes that the linker between two fragments has the flexibility to perform the translation and rotation which is needed for the superposition of the assigned fragments. Additionally, this assumes a comparable length of the linkers in both molecules. Considering these facts, the inherent flexible alignment of the method is a rough approximation, but it increases the ability to do "scaffold-hoppings" without the use of a time-consuming conformational sampling.

A comparison of the OAK with its flexibility extension shows a comparable overall performance at 0.5%. The gap between the methods increases at higher false positive fractions concerning the average rank. In contrast to the average rank, the difference on each data set is marginal with an advantage for the OAK.

As a result, the OAK obtains lower ranks and the difference of the average rank increases. Major differences regarding the performance on each data set can only be seen at 0.5% false positive rate. Surprising is the improved performance of the OAK_FLEX _on the cox2 data set. The inhibitors of the cox2 are usually rigid scaffolds consisting of two or three ring systems. Hence, there should not be a performance gain using an approach that integrates the local flexibility of the atom neighborhood. The active structures of the cox2 data set have terminal substitutions with a depth of two or three bonds. These substitutions form the flexible part of the molecules and have an optimal depth for the local flexibility approach of the OAK_FLEX_. The flexibility information of the terminal substitutions is stored in the atom which is part of the ring system. Therefore, the method has an improved ability to distinguish different substitution stages like ortho, meta and para substitutions. Additionally, the existence of rigid and flexible parts with an ideal length yields a better distinction between rigid and flexible parts of a molecule. The last point was already observed in QSAR experiments reported by Fechner et al. [34] The active structures of the egfr data set contain also a rigid basic scaffold with various substitutions among the molecules. Based on the previous observation, the OAK_FLEX _should attain a higher awROC enrichment as the OAK. Obviously, it is not the case as it can be seen in Table 2. This contradictory result can be explained by the chemical nature of the search query. Erlotinib has two flexible substitutions each with five heavy atoms forming a chain. These substitutions increase the number of rotatable bonds to 11. The average number of rotatable bonds in the active data set of egfr is 4.07. For that reason, the OAK_FLEX _has difficulties to map the 10 flexible atoms of the search query onto the atoms of the active structures. These mappings reduce the overall similarity and performance on the egfr data set. An evidence for this hypothesis is the result on the vegfr2 data set. The search query of the vegfr2 data set has five rotatable bonds, whereas the actives have a mean value of 4.2. Therefore, the penalty of the OAK_FLEX _mapping is reduced and results in a higher enrichment rate, because of the different flexibility properties. The OAAP method achieves good results on the cox2, cdk2, and pdgfrb data set. These results are a consequence of the binning approach on the geometrical distances. The binning represents a fuzzy geometrical distance measure that allows minor changes in the basic scaffold of a structure without reducing the similarity value. This increases the ability to perform "scaffold-hoppings" to related chemotypes with a similar basic structure.

**Figure 7:**
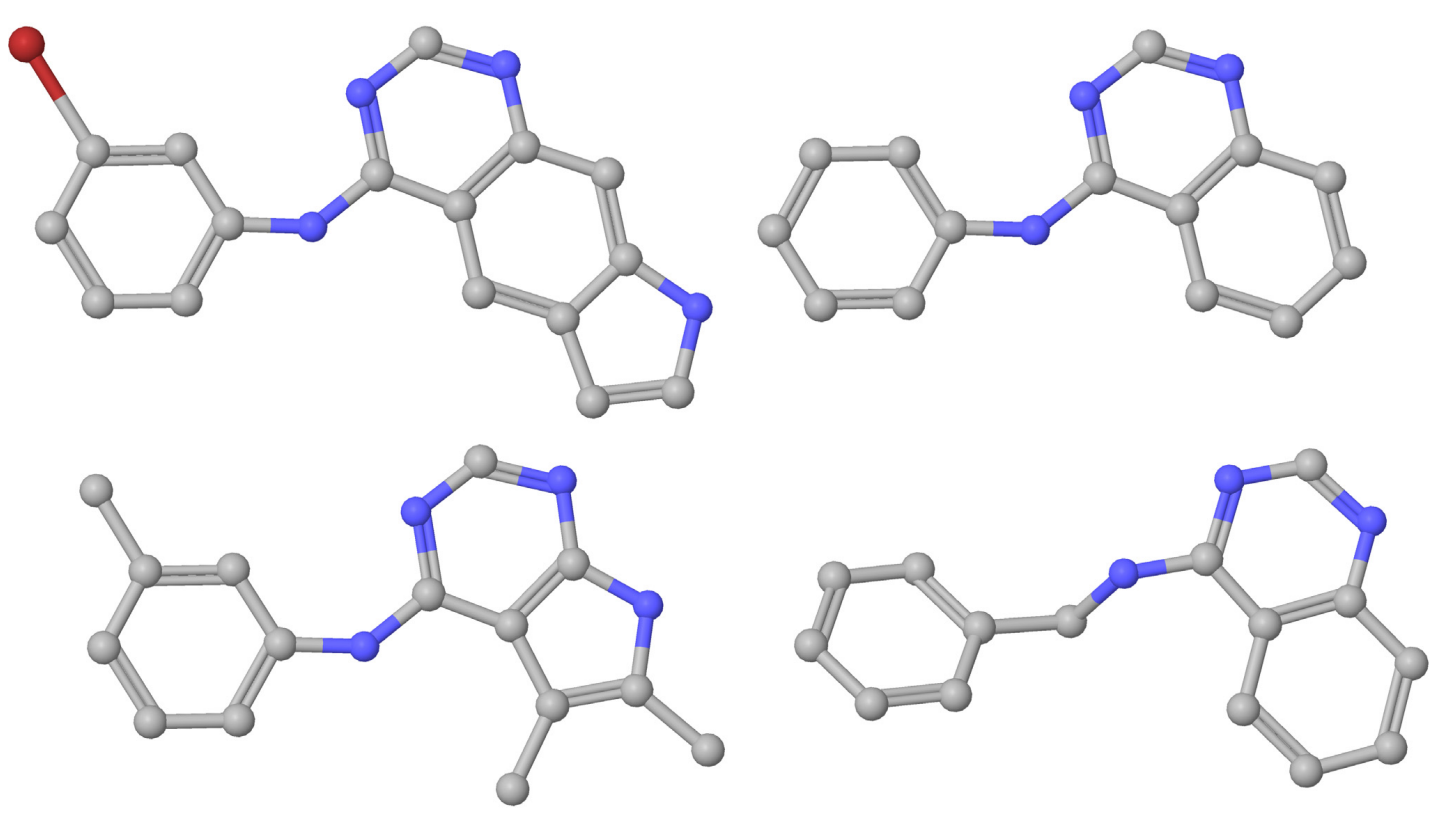
**Example of egfr clusters**. The figure visualizes four parent structures of different egfr clusters. Although these structures belong to different clusters, they share a common basic scaffold.

The awROC enrichment of the 2SHA algorithm at 0.5% on the egfr data set is remarkable. The enrichment on this data set outperforms the results of the other methods. To elucidate this result, we analysed the similarity calculations of the 2SHA on the egfr structures in detail. The active structures have a common property that favors the computation technique of the 2SHA. Each active cluster has a so-called parent molecule. This molecule is the smallest molecule of the cluster regarding the number of heavy atoms [38]. We compared these parent molecules and identified a common basic scaffold, which is included in 32 of 40 clusters and in the search query. Figure 7 shows four examples of this basic scaffold, which consists of a ring system and a condensed heterocyclic system with two or three rings. These systems are aromatic in the query structure and in 25 out of 32 clusters. The 2SHA method identifies the aromatic systems and maps the corresponding systems onto each other in the first assignment step. The superposition of the fragments yields minimal distances of the mapped atoms, because of the planarity of aromatic systems. This results in a high similarity score based on this common basic scaffold and is an explanation of the high awROC enrichment value at 0.5%. Our hypothesis confirms the results of the 2SHA on egfr at higher false positive rates. The method still has the highest enrichment rates, but in comparison to the other methods the difference is significantly reduced.

**Figure 8:**
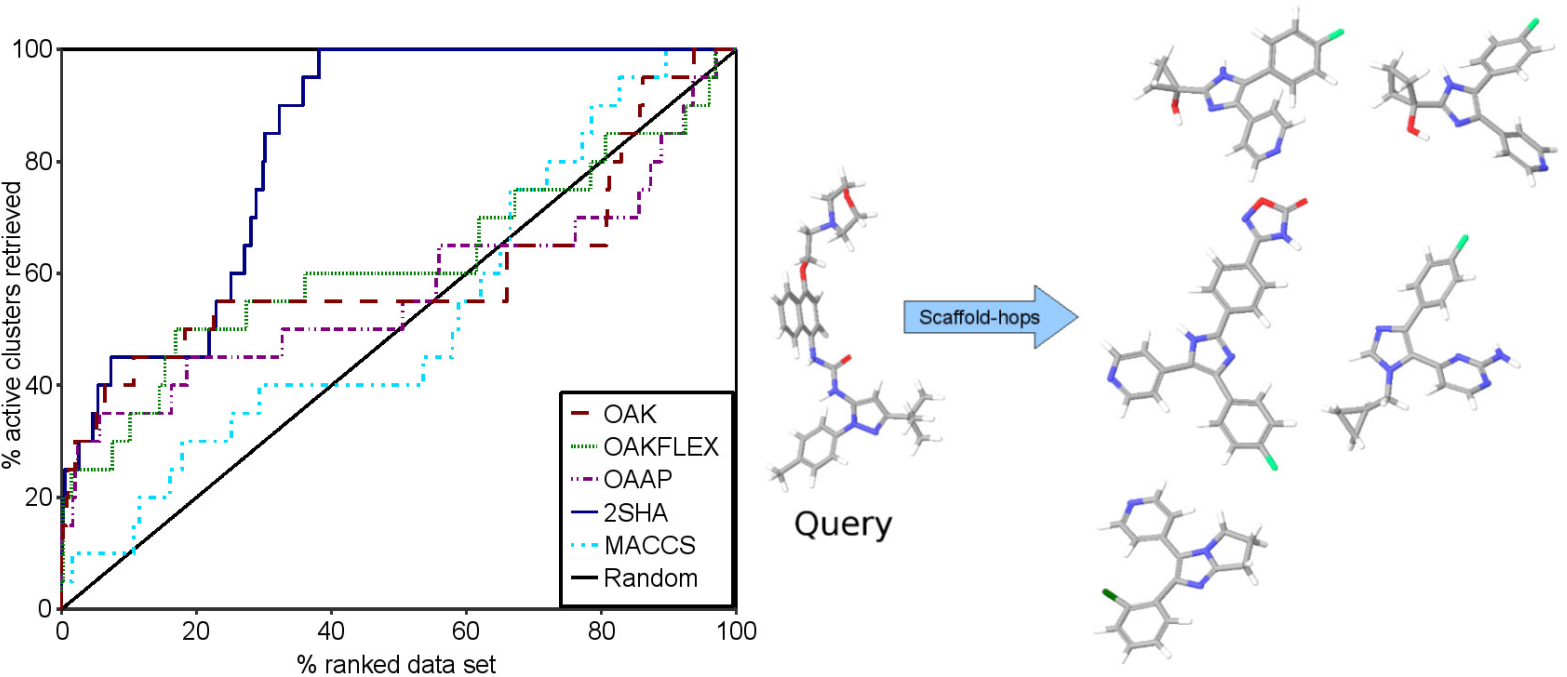
**Chemotype enrichment and "scaffold-hoppings" on p38**. The left figure visualizes the chemotype enrichment of the four optimal assignment methods, the MACCS keys, and the random performance on the p38 data set. A chemotype is considered as retrieved if one structure of the chemotype is ranked. The right figure shows five different chemotypes that were only retrieved by the 2SHA method ranking 25% of the data set.

The awAUC values of Table 6 represent the performance of each method using the complete data set. The 2SHA approach yields the best result with an average rank of 3.08. The FieldScreen method performs better than the remaining optimal assignment techniques OAAP, OAK, and OAK_FLEX_. The DOCK algorithm improves the results and obtained an average rank of 4.27 and achieves a better result than the MACCS keys. Table 6 confirms with the tendency over the awROC enrichments and assigns the best "scaffold-hopping" performance to the 2SHA and FieldScreen. The reduced performance of the other optimal assignment methods can be explained by the results of the chemotype enrichment. Figure 8 shows the chemotype enrichment of the four optimal assignment methods, the MACCS keys, and the random performance on the p38 data set. All optimal assignment methods have a comparable chemotype retrieval until ≈ 50% of the chemotypes are retrieved, which is consistent with the results of the Tables 2, 3, 4, 5. From that point on, the retrieval of the OAK, OAK_FLEX_, and OAAP stagnates and their chemotype enrichment is reduced to the level of the random performance. The reduced enrichment performance of those three optimal assignment methods at higher false positive rates explains the improved results of the FieldScreen approach regarding the awAUC values. Only the 2SHA approach has the ability to achieve a higher generalization of the chemotypes which results in an increased retrieval rate over the complete data set.

### Correlation of Optimal Assignment Methods

**Figure 9:**
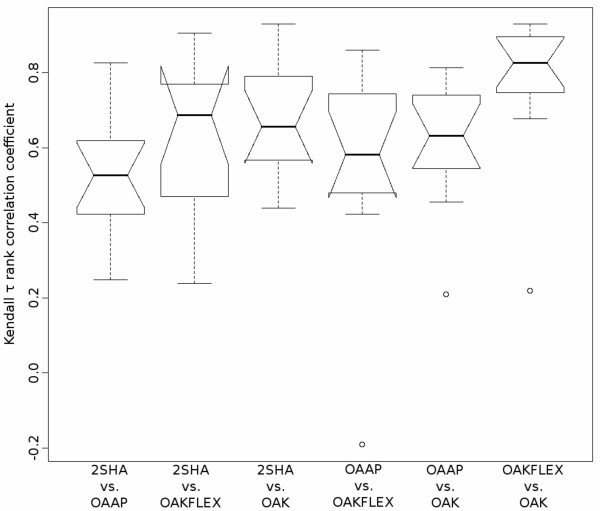
**Rank correlation coefficient of the chemotype discovery between optimal assignment methods**. The diagram illustrates all pairwise rank correlation coefficient of the order of the chemotype discovery between two optimal assignment methods. A high correlation indicates that the order of the chemotype discovery between two methods is similar. Each box plot was created using the correlation of the order of the chemotype discovery on each data set used in this study.

The presented optimal assignment methods are based on the same functional principle. On the one hand, they use different representations and algorithms, but on the other hand, the final similarity computation between two molecules is the result of the assignment algorithm. Hence, it is possible that the two new optimal assignment methods (2SHA and OAAP) are only marginally different compared to the two existing methods (OAK and OAK_FLEX_). The results in the previous section are an evidence that there are differences in the rankings of active and inactive structures. Another interesting question is the order of the chemotype discovery of the individual methods. Different sequences of the chemotype retrieval indicate that the methods explore the chemspace in various directions. Therefore, we analyzed the sequences of all optimal assignment methods on all data sets. We calculated the Kendall's *τ *rank correlation coefficient for each data set to obtain a measure for the correlation of the chemotype retrieval between two methods. This results in 13 correlation coefficients for each pair of methods. The results are illustrated in Figure 9 in form of box plots.

The correlation analysis shows that the 2SHA and OAAP approach have the lowest median of the correlation coefficients. This is the result of a different calculation process to determine the similarity between two atoms. The increased correlation between 2SHA and OAK_FLEX _as well as between 2SHA and OAK can be explained by a common subset of descriptors and the fact that the similarity between two atoms in all three methods is calculated by a RBF. 2SHA has a higher correlation with the OAK_FLEX _than with the OAK. This indicates the already mentioned inherent flexibility consideration of the method. In contrast to the 2SHA, the OAAP method shows a superior correlation to the OAK method without flexibility information. The strong correlation between OAK and OAK_FLEX _is not surprising because the OAK_FLEX _is an extension of the OAK and the standard parametrization of the OAK_FLEX _uses a weight of 0.95 for the OAK similarity [34].

**Figure 10:**
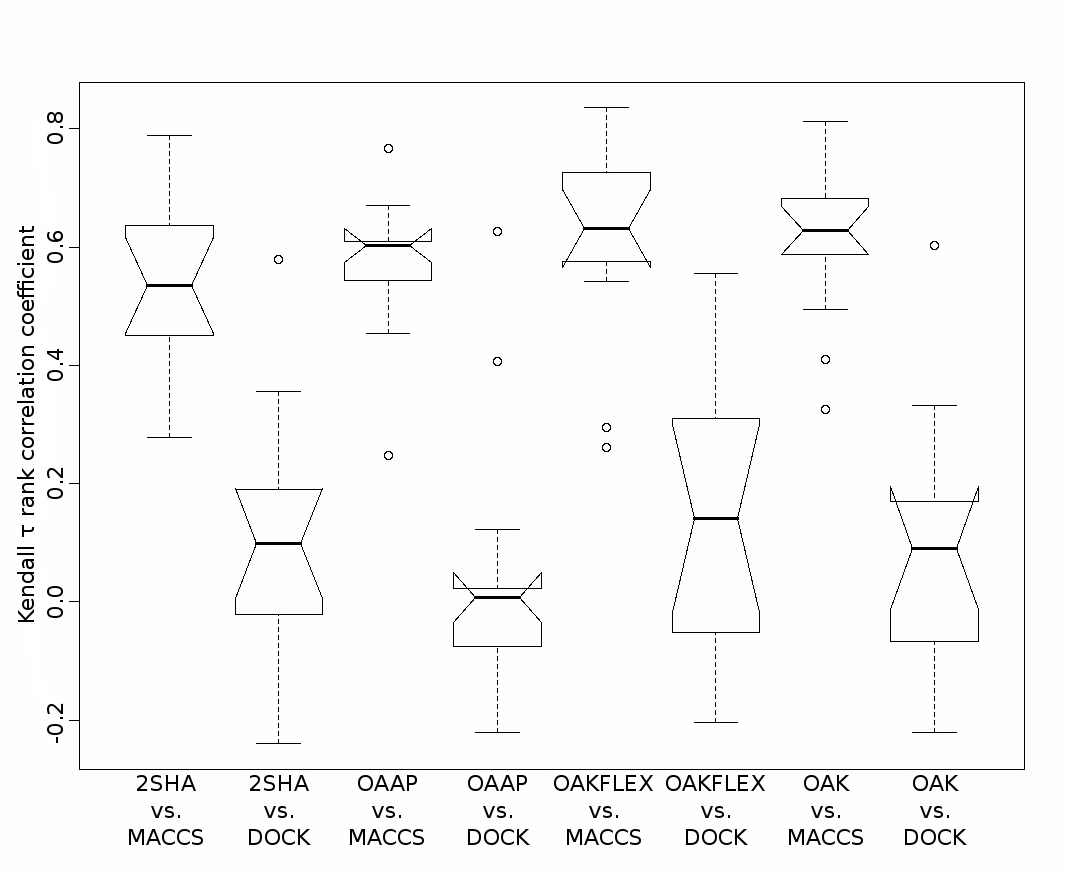
**Rank correlation coefficient of the chemotype discovery between optimal assignment methods and DOCK/MACCS keys**. The boxplots show the correlation coefficients of the order of the chemotype discovery between the optimal assignment methods and DOCK as well as the MACCS keys. The experimental setup is equal to the previous correlation analysis between two optimal assignment methods.

To further analyse the optimal assignment methods and their characteristic behaviour in chemspace, we conducted the same experiment and evaluated the correlation between the optimal assignment methods and DOCK or the MACCS keys. The results can be seen in Figure 10.

The results show that the chemotype retrieval sequence of the optimal assignment methods has no correlation with the sequence retrieved by DOCK. The correlation to the methods considering flexibility information (2SHA and OAK_FLEX_) is marginal increased in comparison to the other methods. This is probably the result of the flexible-ligand method that is used by DOCK allowing the incorporation of the flexibility [53]. The overall low correlation is not surprising given the fundamental differences of ligand-based and docking approaches. The correlation coefficients between the optimal assignment methods and the MACCS keys are comparable to the coefficients in the previous experiment between two optimal assignment methods. From these results, it follows that the similarity of the chemotype retrieval sequence between the MACCS keys and an optimal assignment method is comparable to the similarity of two optimal assignment methods. Therefore, the optimal assignment methods and the MACCS keys explore the chemspace in a comparable direction, but the VS results show that each method has its strengths on different data sets.

The enrichment results of the DOCK algorithm in the Tables 2, 3, 4, 5, 6 show an inferior performance in comparison to the other methods. The findings of the chemotype retrieval sequence disclose an interesting property of DOCK. The uncorrelated sequences demonstrate that DOCK explores the chemspace in an orthogonal manner with respect to the other methods. Accordingly, DOCK retrieves different chemotypes that will only be found by the optimal assignment methods and the MACCS keys if a large part of the data set is ranked.

## Conclusion

We have introduced the concept of the optimal assignment approach in the field of ligand-based VS. We presented two new optimal assignment methods. The OAAP method is based on geometrical distance atom pairs. The 2SHA computes two assignment steps. Each method uses the optimal assignment approach on different types of objects showing the wide field of application of the approach. Another advantage is the interpretability of the mappings using visualizations as shown in Figure 1.

We evaluated the methods on 13 modified DUD data sets covering a wide range of different molecules and chemotypes. In order to grade the results of the optimal assignment approaches we compared the results with a state-of-the-art ligand-based VS method that uses a conformational sampling and molecular fields. Additionally, we provided the results of the 166 bit MACCS keys in combination with the Tanimoto coefficient and the performance of the DOCK algorithm on the same data sets. The results show an improved early enrichment performance of all optimal assignment methods. Analyses show that these early enrichments are the results of the retrievals of chemical similar chemotypes with respect to the search query. With increasing false positive rates this bias is reduced and the gap between the optimal assignment methods and the literature results is successively closed. Only the 2SHA approach has the ability to perform the necessary "scaffold-hoppings" to maintain a robust enrichment and obtained the overall best results. The calculations of the 2SHA method approximate an implicit flexible alignment of the substructures and enables the retrieval of chemotypes that have larger distances to the query in chemspace. Further research will be spent on the 2SHA method in combination with multiple query screenings. The fragmentation and the two assignment steps enable a substructure based data fusion on the fragment level.

The first assignment step can disclose fragment classes with similar fragments of two or more search queries. These classes can be used to enumerate all combinations of the fragments resulting in an increased chemotype coverage. This procedure can further improve the "scaffold-hopping" performance and contribute to the robustness of the method.

The presented methods are fast enough to screen more than one million structures within one day on a single core CPU. This high throughput performance qualifies the presented methods to perform screenings on large databases with the aim to select relevant subsets for a given problem. These subsets can be used in combination with biological screenings or more time-consuming docking algorithms.

## Competing interests

The authors declare that they have no competing interests.

## Authors' contributions

AJ filtered and prepared the DUD data sets, developed the two-step hierarchical assignment approach and the flexibility extension of the Optimal Assignment Kernel, designed the experimental setup, performed the virtual screening experiments, performed the correlation experiments of the methods, drafted the manuscript, and participated in the discussion of the results. GH developed the optimal assignment based on atom pairs, helped to draft the manuscript, and participated in the discussion of the results. NF helped to develop the two-step hierarchical assignment approach and the flexibility extension of the Optimal Assignment Kernel, participated in drafting the manuscript, and participated in the discussion of the results. AZ participated in the development of the original Optimal Assignment Kernel, participated in the design of the experimental setup, and participated in the discussion of the results. All authors read and approved the final manuscript.

## Supplementary Material

Additional file 1 **Complete list atom and bond descriptors of the OAK**. The file OAK_descriptorlist.pdf lists all 32 descriptors of the OAK used in this study. Click here for file

Additional file 2 **Archive of the result files**. The file Results.tar.gz is a Gzip compressed Tar archive containing the results file of each method for each data set. The result files are tab-separated plain text files including the following information: method name, active data set with size, cluster information and the distribution of the molecules over the clusters, decoy data set with size, ratio active:decoy, AUC, awAUC, BEDROC scores for predefined alpha values as suggested by Truchon and Bayly [63], relative enrichment factor [67], ROC enrichments, awROC enrichments at predefined false positive fractions, chemotype enrichment, ROC data points, and the ranking of each structure. Click here for file
