# Supplementary material for: Optimal assignment methods for ligand-based virtual screening
Source: J Cheminform. 2009 Aug 25;1:14. doi: 10.1186/1758-2946-1-14 (PMC2820492; doi:10.1186/1758-2946-1-14)
Supplement: Additional file 1 — Complete list atom and bond descriptors of the OAK. The file OAK_descriptorlist.pdf lists all 32 descriptors of the OAK used in this study. [file 1758-2946-1-14-S1.pdf]

Descriptors of the Optimal Assignment Kernel

descriptors	nominal	numerical
atom	atom in acceptor, atom in donor, atom in don/acc, atom in terminal carbon, atom in aromatic system, atom in ring, atom in conjugated environment, atom is negative, atom is positive, atom is chiral, atom is axial	electrotopological state, partial charge, graph potentials, atom mass, atom electron affinity, atom VanderWaals volume, electrogeometrical state, atom EN (Pauling), intrinsic state, atom free electrons count, atom hybridisation, atom heavy valence, atom implicit valence
bond	bond in aromatic system, bond in ring, bond is rotor, bond is carbonyl, bond is amide, bond is primary amide, bond is ester	Bond length

All descriptors were calculated using JOELib2[1].

1 https://sourceforge.net/projects/joelib/
